# Inhibition of Anti-Inflammatory Macrophage Phenotype Reduces Tumour Growth in Mouse Models of Brain Metastasis

**DOI:** 10.3389/fonc.2022.850656

**Published:** 2022-03-10

**Authors:** Vasiliki Economopoulos, Maria Pannell, Vanessa A. Johanssen, Helen Scott, Kleopatra E. Andreou, James R. Larkin, Nicola R. Sibson

**Affiliations:** Department of Oncology, MRC Oxford Institute for Radiation Oncology, University of Oxford, Oxford, United Kingdom

**Keywords:** neuro-oncology, tumour microenvironment, brain metastasis, microglia, tumour associated macrophages

## Abstract

Breast cancer brain metastasis is a significant clinical problem and carries a poor prognosis. Although it is well-established that macrophages are a primary component of the brain metastasis microenvironment, the role of blood-derived macrophages (BDM) and brain-resident microglia in the progression of brain metastases remains uncertain. The aim of this study, therefore, was to determine the role, specifically, of pro- and anti-inflammatory BDM and microglial phenotypes on metastasis progression. Initial *in vitro* studies demonstrated decreased migration of EO771 metastatic breast cancer cells in the presence of pro-inflammatory, but not anti-inflammatory, stimulated RAW 264.7 macrophages. *In vivo*, suppression of the anti-inflammatory BDM phenotype, specifically, *via* myeloid knock out of Krüppel-like Factor 4 (KLF4) significantly reduced EO771 tumour growth in the brains of C57BL/6 mice. Further, pharmacological inhibition of the anti-inflammatory BDM and/or microglial phenotypes, *via* either Colony Stimulating Factor 1 Receptor (CSF-1R) or STAT6 pathways, significantly decreased tumour burden in two different syngeneic mouse models of breast cancer brain metastasis. These findings suggest that switching BDM and microglia towards a more pro-inflammatory phenotype may be an effective therapeutic strategy in brain metastasis.

## 1 Introduction

Brain metastasis is a devastating diagnosis for patients with primary breast cancer, and the prognosis is extremely poor; patient survival ranges from 2 to 23 months from diagnosis (1, 2). Many gaps exist in our knowledge regarding the pathogenesis of brain metastasis, but the role of the microenvironment, and cells of the innate immune system (monocytes/macrophages) in particular, remain a subject for debate.

In extracranial metastases, macrophages have been shown to promote disease progression. Gil-Bernabé et al. demonstrated that blood-derived macrophages are associated with arrested tumour cells in the lung, and that depletion of myeloid cells in the transgenic CD11b-DTR mouse decreases tumour cell survival and prevents the establishment of micrometastases (3). Similarly, Qian et al. showed that depletion of the macrophage population, using clodronate liposomes, hinders the establishment of metastases in the lung (4). More specifically, Yao et al. found that inhibiting anti-inflammatory (M2) macrophage polarization, with the tyrosine kinase inhibitor imatinib, significantly reduced the number of nodules present in the lungs in a lung cancer model, in part due to STAT6 inhibition (5). Similarly, Binnemars-Postma et al. evaluated STAT6 inhibition *in vivo* and showed that inhibition in macrophages reduces tumour growth and development of the metastatic niche within the liver in a murine breast cancer model (6).

Given its unique environment, however, the role that macrophages are known to play in extracranial metastases and primary tumours may not be reflected in the brain. Nevertheless, there is evidence to suggest that the role of macrophages may be similar. In models of glioma, macrophage inhibition through targeting Colony Stimulating Factor-1 Receptor (CSF-1R) decreases tumour volume (7, 8). Since the CSF-1R pathway promotes anti-inflammatory M2 activation and expansion of macrophages and microglia (9–11), it seems likely that the phenotype of these macrophages/microglia is an important factor in brain tumour growth. Indeed, work by Rippaus et al. demonstrated that parenchymal brain metastases have a more anti-inflammatory M2-like phenotype (12), whilst recent work by Andreou et al. demonstrated that specific depletion of anti-inflammatory M2-like macrophages/microglia, using mannosylated clodronate liposomes, in a breast cancer brain metastasis model resulted in decreased tumour burden (13). However, there is also evidence to suggest that macrophages may have an anti-tumour effect; Galarneau et al. found that overall depletion of myeloid cells in CD11b-TK mice treated with ganciclovir actually increased glioma growth (14). Moreover, it is now well established that the dichotomous M1/M2 macrophage polarization paradigm does not fully capture the complexity of the macrophage/microglial activation states, especially when these cells are associated with tumours and experience a wide variety of pro-inflamamtory (M1) and anti-inflammatory (M2) stimuli simultaneously (10). Throughout this work, therefore, we will rather refer to M1 phenotypes and stimuli as pro-inflammatory, and M2 phenotypes and stimuli as anti-inflammatory.

Overall, therefore, uncertainty remains as to the role of macrophages/microglia in both primary and secondary brain tumours, although evidence points towards a pro-tumorigenic role for the predominantly anti-inflammatory polarised subpopulation. Further, it is unclear as to whether brain resident microglia and blood-derived macrophages recruited to brain tumours play differential roles in tumour progression. The primary aims of this study, therefore, were (i) to determine whether anti-inflammatory blood-derived macrophages (BDM) promote tumour cell migration *in vitro*, (ii) to determine whether there is significant infiltration of anti-inflammatory BDM into the microenvironment of breast cancer brain metastases *in vivo*, (iii) to determine whether suppression of anti-inflammatory BDM activation through genetic knock-out reduces brain metastasis growth, and (iv) whether pharmacologically inhibiting BDM and/or microglial populations, or switching them to a more pro-inflammatory phenotype, reduces brain metastasis growth.

## 2 Materials and Methods

### 2.1 Cell Culture

The EO771 cell line (mouse metastatic medullary mammary carcinoma, C57BL/6 background (15), kindly provided by Prof. Mihaela Lorger, University of Leeds) was cultured in RPMI-1640 medium with 20% FBS, 1% L-Glutamine, 1% non-essential amino acids and 1% sodium pyruvate. The 4T1-GFP cell line [mouse metastatic mammary carcinoma, BALB/c background (16)] was purchased from ATCC and cultured in DMEM medium with 10% FBS and 1% L-Glutamine. The RAW 264.7 cell line (mouse macrophage, kindly provided by Prof. Xin Lu, University of Oxford) was cultured in DMEM medium with 10% FBS and 1% L-Glutamine. All cells were maintained at 37°C and in 5% CO_2_.

### 2.2 Effect of Macrophage Phenotype on Tumour Invasion

RAW 264.7 cells were seeded into the bottom portion of 24-well Matrigel Transwell plate system (Corning) and allowed to adhere overnight. These cells were then treated with either the pro-inflammatory stimulus lipopolysaccharide (LPS; 10 µg/mL) or the anti-inflammatory stimulus interleukin-4 (IL-4; 20 ng/mL), or left untreated as a control for 24 hours. Subsequently, EO771 cells were seeded into the top portion of the Transwells, in wells containing either RAW cells or just the treatment media in the bottom portion. Invasion of the EO771 cells was measured 24 hours later by counting the number of cells that had crossed through the Transwell membrane normalized to the total number of cells on both the upper and lower sides of the Transwell insert (n = 6 for each condition).

The phenotype of the stimulated RAW 264.7 cells was assessed by immunofluorescent staining for the pro-inflammatory marker inducible nitric oxide synthase (iNOS) and the anti-inflammatory marker arginase 1 (Arg1), after 24 hours incubation with either LPS (10 µg/mL), IL-4 (20 ng/mL) or control media. Normalized staining areas were calculated by dividing the measured stained area of the marker by the number of nuclei present, as determined through DAPI staining. The ratio of normalized iNOS stained area to normalized Arg1 stained area was then calculated to determine the predominant phenotype of the stimulated cells.

### 2.3 Intracerebral Models of Brain Metastasis

All animal experiments were assessed by the University of Oxford Clinical Medicine Ethics Review Committee and approved by the UK Home Office (Animals [Scientific Procedures] Act 1986), and conducted in accordance with the University of Oxford Policy on the Use of Animals in Scientific Research, the ARRIVE Guidelines and the Guidelines for the Welfare and Use of Animals in Cancer Research (17). All mice were maintained in a specific pathogen free environment and transgenic mice were obtained from in-house breeding colonies.

Mice were injected intracerebrally with EO771 cells in PBS into the left striatum, as described previously (18, 19). Briefly, mice were anaesthetised using 3% isoflurane in 30% oxygen and 70% nitrous oxide, mounted on a stereotactic frame and, subsequently, maintained at 2% isoflurane during surgery. An incision was made on the top of the scalp to expose the skull and a burr hole drilled 0.5 mm forward and 2 mm to the left of bregma. A pulled glass microcapillary (tip <75μm) was inserted stereotaxically through the burr hole to a depth of 2.5 mm from the surface of the brain. EO771 cells (500 cells in 0.5 μl) were injected over 5 min, and the microcapillary left in place for a further 5 min before withdrawing slowly and the scalp wound sutured. Animals were excluded from the study if evidence of extracranial tumour development was found during the course of the experiment. Animals were also excluded if the tumour was found on histological examination to have been injected within the ventricle. Pilot studies were conducted to optimize this injection model.

In order to be able to study a longer time-course of metastasis growth in the brain, it is necessary to use this intracerebral induction model rather than a systemically induced model, as the latter results in significant systemic metastasis burden and animal welfare issues; consequently, longer-term studies are precluded. Previous work by Serres et al. has demonstrated that intracerebral induction route produces similar growth patterns of metastatic colonies and inflammatory responses as an intracardiac injection model, and also recapitulates the microenvironment of human brain metastases (20). Whilst the intracerebral injection model used in that particular study was in rats, similar features are found in the mouse intracerebral injection models used in the current study.

#### 2.3.1 Assessment of Blood-Derived Monocyte/Macrophage Recruitment to Brain Metastases *In Vivo*

Female Lys-GFP-ki transgenic mice (C57BL/6J background; 7-9 weeks) were used to assess recruitment of blood-derived macrophages (BDM) to the brain, as this strain possesses GFP expression in myeloid cells, but not microglia (21, 22). Mice were injected intracerebrally with EO771 cells, as described above, at day 0 and were perfusion-fixed 7, 14 or 21 days later for histological assessment (details below).

#### 2.3.2 Suppression of Anti-Inflammatory Phenotype in Blood-Derived Monocytes/Macrophages

LysM^Cre/Cre^KLF4^fl/fl^ (C57BL/6J background; 7-9 weeks, kindly provided by Dr Xudong Liao, Case Western Reserve University) transgenic mice were used to assess the effect of BDM phenotype on brain metastasis growth. In these mice, the KLF4 transcription factor is knocked out in BDM (23), resulting in suppression of the anti-inflammatory phenotype through downstream inhibition of the STAT6 pathway. For these experiments, LysM^Cre/Cre^ mice of the same background were used as controls. Blood-derived specificity of the KLF4 knock out was confirmed through genotyping of primary microglia and BDM isolated from the bone marrow of LysM^Cre/Cre^KLF4^fl/fl^ mice (**Figure S1**). Mice were injected intracerebrally, as above, with EO771 cells and perfusion-fixed 21 days later for histological assessment.

#### 2.3.3 Pharmacological Inhibition of Macrophages/Microglia *In Vivo*

To further assess the roles of BDM/microglia, and the anti-inflammatory phenotype specifically, Lys-GFP-ki mice (as above) were treated with either macrophage inhibitory peptide (Tuftsin fragment 1-3, TKP, Bachem) or the CSF-1R neutralizing antibody (M279, Amgen). TKP has previously been shown to reduce overall macrophage/microglial activation, and to shift their transcriptional profile to more anti-inflammatory (Th2) in experimental autoimmune encephalomyelitis and spinal cord injury models (24, 25). In contrast, M279 has been shown to suppress the anti-inflammatory macrophage expansion (26) and to exert its effects on the CSF-1R pathway exclusively in BDM, and not brain-resident microglia (27). Control animals were treated with PBS. All treatments were delivered by osmotic minipump (100 µL 28 day release, model 1004 Alzet Osmotic Minipump, purchased through Charles River) which delivers its payload at a rate of 0.11µL/hour.

One week prior to intracerebral injection, animals underwent surgery to implant the minipump. Animals were anaesthetised with 3% isoflurane in 30% oxygen and 70% air, and subsequently maintained with 2% isoflurane. Immediately prior to surgery preparation, animals received a subcutaneous injection of Vetergesic (Buprenorphine, 0.1 mg/kg) and a local injection of Marcaine 0.25% (bupivacaine, 7.5 mg/kg) at the incision/implantation site. The fur at the back of the neck and on the upper back was then clipped and cleaned with a chlorhexidine preparation. An incision was made just below the base of the neck from the left to right of the animal and a sufficiently sized pocket was created under the skin on the back through the incision. A filled pump was placed nozzle-end first into the pocket, and a suture placed internally to secure the pump in the pocket. The incision was sutured closed.

After a 48 hour *in vivo* priming period, mice were treated for 5 days with either TKP (2.27 mM in PBS), M279 (50 mg/mL in PBS) or PBS alone, prior to cell injection. At day 7, mice were injected intracerebrally, as above, with EO771 cells, and were perfusion-fixed at day 28 post-minipump implantation (21 days post-cell injection) for histological assessment (details below).

### 2.4 Inhibition of Anti-Inflammatory Macrophages/Microglia in an Intracardiac Brain Metastasis Model

To assess the effects of inhibiting the anti-inflammatory BDM/microglia phenotype on brain metastasis development, a model that more closely mimics the natural development of brain metastases was used. Female BALB/cAnNCrl mice (7-9 weeks old; Charles River, Margate, UK) were injected with 10^5^ 4T1-GFP cells in 100 µL of PBS *via* the left ventricle of the heart under ultrasound image guidance, as described previously (28), to allow haematogenous dissemination to the brain. One week after intracardiac injection, animals were implanted with subcutaneous osmotic minipumps, as above. Following 48 hours *in vivo* priming, mice were treated with either the STAT6 inhibitor AS1517499 (11.43 mg/mL; 10.6 mg/kg/week) or vehicle (50% DMSO in water) for 7 days from day 9 after intracardiac injection. AS1517499 is BBB penetrant (29) and, thus, will inhibit the STAT6 pathway in both BDM and microglia. Animals were perfusion-fixed at 16 days post-intracardiac injection and prepared for histological assessment (detailed below).

### 2.5 Immunofluorescence

For immunofluorescent analysis of macrophage/microglial infiltration and phenotype, animals were deeply anaesthetised with sodium pentobarbital (40 mg/mL; 0.2 mL injected i.p.) and transcardially perfusion-fixed with heparinised saline, followed by periodate-lysine-paraformaldehyde with 0.25% glutaraldehyde (PLP_light_). The brains were removed, cryopreserved in 30% sucrose and frozen in OCT. Tissue sections were cut at a thickness of 10 µm and allowed to dry on the slides for 24 hours.

Sections were stained for a variety of antigens as either single, double or triple immunofluorescent stains. The following antigen targets were stained (see **Table S1** for antibody details): iNOS (rabbit anti-mouse) with Arg1, F4/80 with iNOS (rabbit anti-mouse), F4/80 with Arg1, TMEM119 with F4/80 and iNOS (mouse anti-mouse), TMEM119 with F4/80 and Arg1. In the case of iNOS two different antibodies were used as differentiated above. TMEM119 staining was used to differentiate microglia from BDM in the KLF4 knock out study (30–35). Using Lys-GFP-ki mice, we confirmed that there was minimal TMEM119 and GFP colocalization (**Figure S2**).

### 2.6 Fluorescent Image Acquisition and Analysis

Images of entire brain sections for volume calculation were acquired using a Nexelom Celigo image cytometer (Nexelom, Manchester, UK) using the slide scanning function. Cell culture plates were also imaged and analysed with this system using the expression analysis function. Tumour foci were identified and measured using DAPI stained sections.

In the intracardiac 4T1 model, DAPI stained images were used to measure tumour burden, which was calculated by measuring the total area of metastases within sections and then dividing by the total section area of analysed sections. The number of lesions per area was measured by dividing the number of metastases measured by the total section area of analysed sections. Average lesion area was calculated by dividing the total area of lesions by the total number of lesions.

All confocal images were acquired on a Zeiss LSM 710 inverted confocal microscope. Images were acquired as z-stacks with 10 slices and a spacing of 1.46 µm using a 20x objective, a software zoom of 1 and with 4 averages. Images were acquired from at least 3 separate tissue sections spaced 60 - 180 µm apart for each animal.

Co-localization analysis was performed on confocal images between immunostained markers and GFP using an automated in-house ImageJ plugin. All analyses were performed on raw, unprocessed images. In this analysis, the threshold for positive signal for each marker was defined as the average signal of the background plus 3 standard deviations, as determined from images specifically acquired of the background signal. The plugin calculated the areas of positive signal based on these thresholds for each marker, whilst tracking the area of co-localized pixels for each combination of markers present in the image. The co-localization between markers was calculated as the percentage of marker A area that co-localized with marker B, by dividing the area of co-localized pixels (A + B) by the total area of marker A. All calculations were performed by the automated inhouse ImageJ plugin with the calculated thresholds for each marker being fed into this software.

The colour thresholding tool was used to quantify the number of cells that were GFP+ and either F4/80+ or F4/80- using previously calculated thresholds. First, the GFP+ threshold was used to create a mask of all GFP+ cells and this was then used to block out any areas that were not GFP+. Next the F4/80+ threshold was used to create a second mask that highlighted areas of F4/80+ staining. Finally, the masks were overlapped to identify areas of GFP and F4/80 co-localization, and the number of cells that fell into the GFP+F4/80+ or GFP+F4/80- categories were counted.

### 2.7 Statistical Analysis

Sample sizes required for sufficient statistical power were calculated using pilot data. The details of these calculations are shown in **Table S2** and were performed using the methodology described by Kadam and Bhalerao (36). Analysis was performed on measurements taken from independent samples.

In the KLF4 knock out study, a two-tailed Student’s t-test was used to compare tumour volume, cell infiltration and cell phenotype between the control and knockout groups. In the characterization study and the pharmacological inhibition study (TKP and M279), expression of histological markers was compared using a one-way ANOVA test, with Welch’s ANOVA used where the standard deviation of data varied significantly between groups. The Tukey’s and Dunnett’s (Welch’s ANOVA) post-tests were used when ANOVA results were significant, to compare individual groups. A two-way ANOVA was used to compare iNOS and Arg1 expression over time in the characterization study.

In the intracardiac 4T1-GFP model, a two-tailed Student’s t-test was used to compare tumour burden, number of lesions/area and average lesion area between the AS1517499 treated and vehicle only treated groups. A two-tailed Student’s t-test was also used to compare the expression of immunostained markers in both the KLF4 knock out model and the intracardiac 4T1-GFP model.

Normality testing was performed using the Shapiro-Wilk test and data that were found to not follow a normal distribution in all experiments were analysed using non-parametric versions of the above tests (Mann-Whitney and Kruskal-Wallis tests). All sample sizes for groups in each study are reported within figure captions.

To ensure data reproducibility, we monitored the volume of the control groups in each experiment to ensure that these remained similar. Animals that received the various pharmacological treatments were distributed as evenly as possible between all cages to ensure that both control and treatment variability could be controlled. The researcher was blinded to treatment group and knock out status of animals during the analysis of the data, and animals were identified in a manner that only the particular experiment they were in could be known.

## 3 Results

### 3.1 Effect of Macrophage Phenotype on Tumour Cell Invasion *In Vitro*

**Figure 1:**
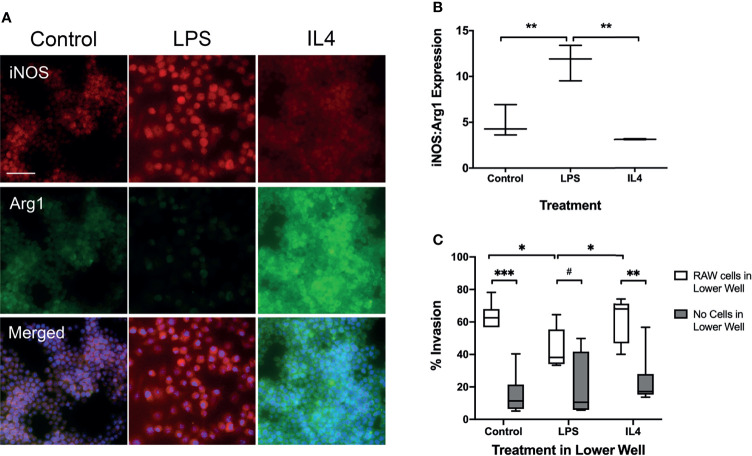
Transwell migration assay with RAW 267.4 macrophages and EO771 metastatic tumour cells. **(A)** Immunofluorescent staining of pro-inflammatory marker iNOS and anti-inflammatory marker Arg1 in macrophages to assess phenotype following incubation with either the pro-inflammatory stimulus LPS or the anti-inflammatory cytokine IL4. Bottom panel of images also contains nuclear (DAPI) counterstain (blue). **(B)** Graph showing ratio of iNOS : Arg1 expression in macrophages treated with either LPS (n = 6) or IL4 (n = 6), compared to untreated macrophages (n = 6). **(C)** Graph showing percentage of EO771 tumour cells migrating through the Matrigel coated Transwell membrane in the presence of either LPS (n = 6) or IL4 (n = 6) stimulated or untreated (n = 6) macrophages (white bars), or the stimulus media alone without macrophages (grey bars). Data shown as box and whisker plots depicting full range of data points. Scale bar = 100 µm. *p < 0.05, **p < 0.01, ***p < 0.001, ^#^p = 0.0756”.

To determine whether macrophage phenotype alters tumour cell invasion *in vitro*, cultured macrophages were treated with either LPS to induce a pro-inflammatory phenotype or IL-4 to induce an anti-inflammatory phenotype. The phenotype of LPS stimulated, IL-4 stimulated and unstimulated control RAW264.7 cells was confirmed by determining the ratio of iNOS : Arg1 expression immunofluorescently (**Figure 1A**). Since the phenotype of macrophages exists as a continuum, rather than as discrete classes, the ratio of iNOS : Arg1 was used in addition to individual expression levels to assess shifts in macrophage and microglial phenotype. LPS stimulated cells showed a significantly higher iNOS : Arg1 ratio than control cells (**Figure 1B**; p < 0.01), indicating a predominantly pro-inflammatory phenotype. Conversely, the IL-4 stimulated cells showed a significantly reduced iNOS : Arg1 ratio compared to LPS stimulated cells (p < 0.05), indicating a more anti-inflammatory phenotype, although the ratio was not significantly different to control cells (p = 0.1478). In the Transwell invasion assay, significantly fewer EO771 cells passed through the Matrigel membrane in the wells with LPS stimulated macrophages in the bottom chamber, compared to both IL-4 stimulated and control macrophages (**Figure 1C**; p < 0.05). Increased EO771 invasion was also evident in IL4 stimulated (p < 0.01) and control (p < 0.001) wells where RAW macrophages were present, compared to the media + stimulus only wells (**Figure 1C**).

### 3.2 Infiltration of Blood-Derived Monocytes/Macrophages *In Vivo*

**Figure 2:**
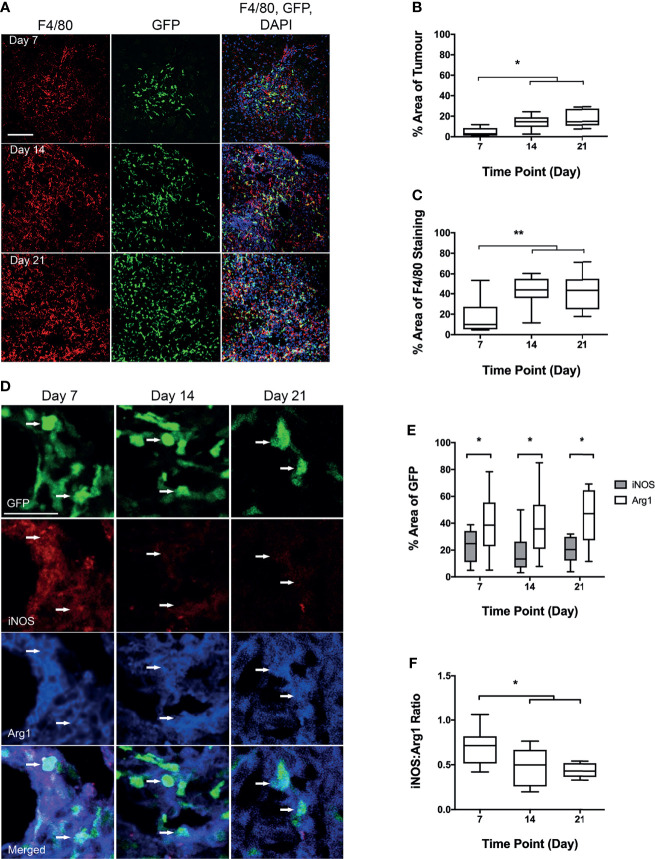
Analysis of blood-derived macrophage (BDM) infiltration and phenotype into tumour foci from Lys-GFP-ki mice injected intracerebrally with EO771 cells. **(A)** Co-localisation of GFP fluorescence (BDM; green) with immunofluorescent staining for F4/80 (all microglia/macrophages; red). Nuclei stained with DAPI (blue). Scale bar = 100 µm. **(B)** Graph showing percentage of tumour area that is GFP positive indicating infiltration of BDM. **(C)** Graph showing percentage of F4/80 staining that is GFP positive, indicating relative proportions of BDM and brain-resident microglia within the tumour foci. **(D)** Co-localisation of immunofluorescent staining for iNOS (pro-inflammatory; red) or Arg1 (anti-inflammatory; blue) with GFP in BDM. Arrows indicate GFP+ BDM with colocalization of iNOS and Arg1. Scale bar = 30 µm. **(E)** Graph showing percentage of GFP-positive area that is either iNOS (grey bars) or Arg1 (white bars) positive. **(F)** Graph showing ratio of iNOS : Arg1 in GFP-positive cells. N = 9 at day 7, n = 9 at day 14 and n = 10 at day 21. Data shown as box and whisker plots depicting full range of data points. *p < 0.05, **p < 0.01.

Infiltration of BDM into brain metastases *in vivo*, was assessed in the Lys-GFP-ki transgenic mice (**Figure 2A**), in which GFP is expressed in myeloid cells and not within microglia (21, 22). The percentage of GFP within metastasis foci in the brain increased significantly from day 7 to days 14 and 21 (p < 0.001; **Figure 2B**). Whilst overall expression of the mouse macrophage/microglial marker F4/80 as a percentage of tumour area did not change significantly, the percentage of F4/80 expressing cells that were also GFP positive increased significantly from day 7 to days 14 and 21 (p < 0.01; **Figure 2C**). The GFP positive area of the tumours correlated with tumour volume (p < 0.0001; **Figure S3**). The proportion of GFP positive cells that were also F4/80 positive was greater than 90% on average, indicating that they were predominantly monocytes/macrophages, and did not vary significantly between time points (**Figure S2A**). Interestingly, qualitative observation suggested that microglial recruitment was greater at the margins of the tumours, whilst BDM were evident throughout.

### 3.3 Immune Phenotype of Infiltrating Blood-Derived Monocytes/Macrophages

Tissue sections stained for both Arg1 and iNOS (**Figure 2D**), showed significantly higher levels of Arg1 co-localisation with GFP positive cells, than for iNOS (p < 0.05; **Figure 2E**). The ratio of iNOS/Arg1 on GFP positive cells decreased significantly from day 7 to days 14 and 21 (p < 0.05; **Figure 2F**), indicating an increasingly anti-inflammatory phenotype in recruited blood-derived myeloid cells, predominantly monocytes/macrophages (see above).

### 3.4 Suppression of Anti-Inflammatory Phenotype in Blood-Derived Monocytes/Macrophages

**Figure 3:**
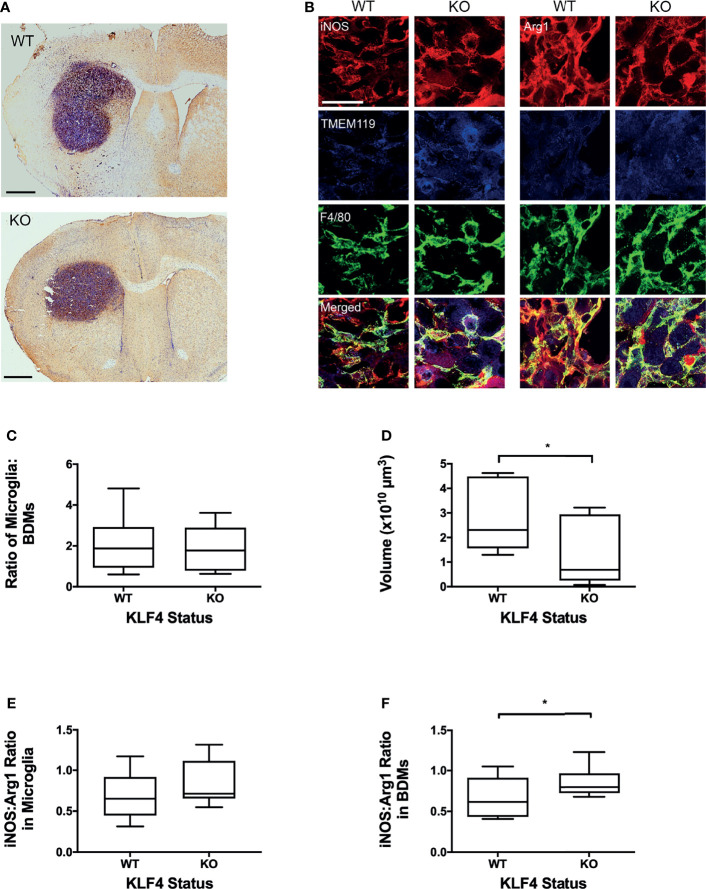
Effect of KLF4 knock out in myeloid cells on phenotype of blood-derived macrophages (BDM) and microglia in brain metastases and tumour growth. **(A)** Representative IHC images showing F4/80 staining in metastases for wild-type (upper panel, n = 9) and knock-out (lower panel, n = 8) mice. Sections counterstained with cresyl violet. Scale bars = 1 mm. **(B)** Immunofluorescent staining of F/480 (total macrophage/microglia; green), TMEM119 (microglia; blue) with either iNOS (left panels, red) or Arg1 (right panels, red) in WT and KLF4-KO mice. Scale bar = 50 µm. **(C)** Graph showing ratio of microglia (TMEM119+ F4/80+) to BDM (TMEM- F4/80+) in WT and KLF4-KO mice. **(D)** Graph showing tumour volumes in WT and KLF4-KO mice. **(E, F)** Graphs showing ratios of iNOS : Arg1 expression, in WT and KLF4-KO mice, in **(E)** microglia (p = 0.152) and **(F)** BDM (p = 0.036). Data shown as box and whisker plots depicting full range of data pointspoints. *p < 0.05.

Next, to determine the effect of anti-inflammatory BDM, specifically, on tumour growth, myeloid specific deletion of KLF4 was used to suppress STAT6-mediated anti-inflammatory macrophage activation (**Figure 3**). Representative images of tumours stained for F4/80 are shown in **Figure 3A**. Immunofluorescent images stained with F4/80 and TMEM119 with both iNOS and Arg1 are shown in **Figure 3B**. No significant differences were found between control and KLF4 knockout (KLF4-KO) mice for either the percentage of tumour area showing overall macrophage/microglia infiltration (F4/80 staining) or, specifically, microglial infiltration/activation (TMEM119+F4/80+) or BDM infiltration (TMEM119-F4/80+; **Figure S5**). The ratio of BDM to microglia was not significantly different between WT and KO animals (**Figure 3C**). A significant reduction in tumour volumes, however, was evident in the KLF4-KO group compared to control animals (p < 0.05; **Figure 3D**). In the TMEM119 positive microglial population, no significant difference was found between KLF4-KO and controls for either Arg1 or iNOS expression (**Figure S4**), and the ratio of iNOS : Arg1 was not significantly different (**Figure 3E**). In contrast, the level of Arg1 expression was significantly reduced in BDM in the KLF4-KO group (p < 0.05), whilst the level of iNOS expression remained unchanged (**Figure S4**). Consequently, the iNOS : Arg1 ratio in BDM increased significantly in the KLF4-KO group (p < 0.05; **Figure 3F**).

### 3.5 Inhibition of Macrophages/Microglia *In Vivo*

**Figure 4:**
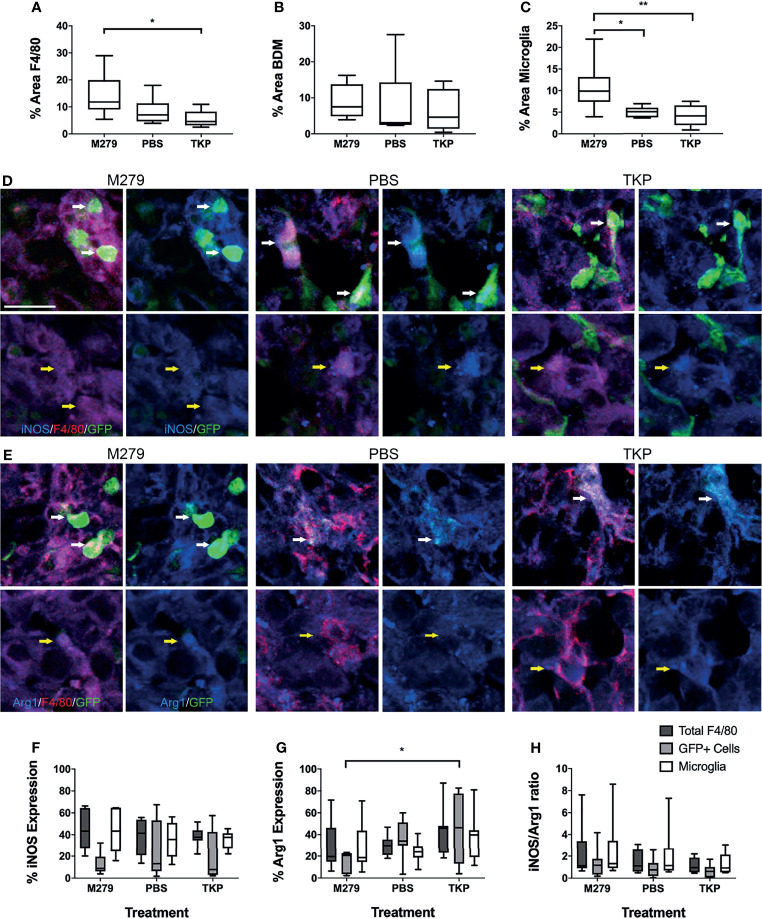
Effect of phenotype modulation of microglia and blood-derived macrophages (BDM) with TKP and the CSF-1R neutralizing antibody, M279. **(A–C)** Graphs showing percentage of tumour area that is stained for **(A)** all F4/80+ cells, **(B)** GFP+ BDM and **(C)** GFP- microglia. **(D, E)** Immunofluorescent staining of F4/80 (red) with **(D)** iNOS or **(E)** Arg1 (blue) and endogenous GFP in M279 (n = 7), PBS (n = 7) and TKP (n = 8) treated mice. In each panel, the upper rows are highlighting GFP+ BDM (white arrows), whilst the lower rows are highlighting GFP- microglia (yellow arrows). **(F–H)** Graphs showing expression of **(E)** iNOS, **(F)** Arg1 and **(G)** ratio of iNOS : Arg1 expression in all F4/80+ cells (dark grey bars), GFP+ BDM (light grey bars) and microglia (white bars). Data shown as box and whisker plots depicting full range of data points. Scale bar = 20 µm. *p < 0.05.

**Figure 5:**
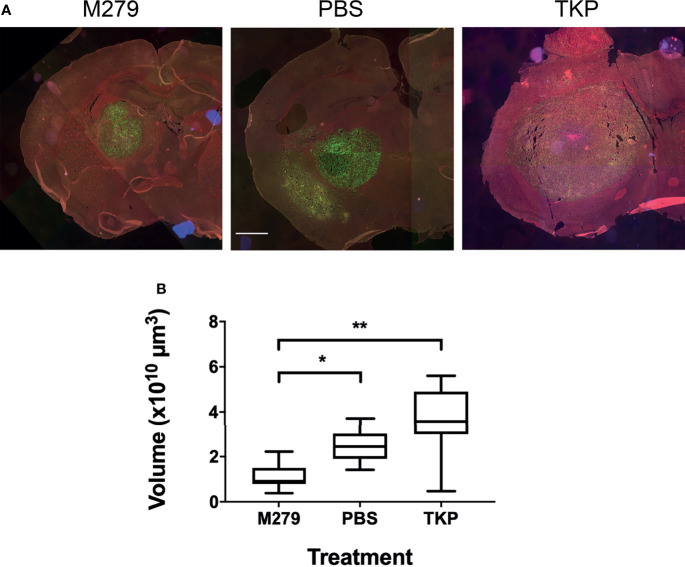
Effect of TKP and M279 treatment on tumour volume. **(A)** Representative images of brain sections from M279 (CSF-1R mAb), PBS and TKP treated mice. Scale bar = 1 mm. **(B)** Graph of tumour volumes in M279 (n = 7), PBS (n = 7) and TKP (n = 8) treated groups. Data shown as box and whisker plots depicting full range of data points. *p < 0.05, **p < 0.01.

We next assessed the effects of inhibiting pro- and anti-inflammatory BDM/microglial phenotypes through TKP and M279 treatment, respectively. Again, the Lys-GFP-ki mouse strain was used, to enable differentiation between BDM and brain-resident microglia. Immunofluorescent staining for F4/80, iNOS and Arg1 demonstrated changes in infiltration and phenotype in the BDM and microglial populations according to treatment (**Figure 4**). Overall levels of F4/80 staining within tumours was significantly higher in mice treated with M279 (CSF-1R mAb) compared to TKP treatment mice (p < 0.05; **Figure 4A**). Whilst no differences were observed between groups for GFP positive myeloid cell infiltration (**Figure 4B**), microglial infiltration was significantly higher in M279 treated mice compared to both PBS and TKP treated mice (p < 0.05; **Figure 4C**). The proportion of GFP positive cells that were also F4/80 positive did not vary between treatment groups and remained above 90% as for the previous study, indicating that the majority of recruited myeloid cells were BDM (**Figure S2B**). Colocalization analysis of iNOS expression (**Figure 4D**) and Arg1 (**Figure 4E**) demonstrated changes in phenotype of BDM. iNOS expression did not vary significantly between groups in any macrophage/microglial population (**Figure 4F**). In contrast, Arg1 expression in GFP positive BDM was significantly lower in M279 treated mice compared to the TKP treated group (p < 0.05; **Figure 4G**). Although, there appeared to be a trend towards a reduction in iNOS : Arg1 in all populations from the M279 treated group compared to the TKP treated group (**Figure 4H**), the ratio of iNOS : Arg1 expression was not significantly different between groups in any cell population, likely owing to inter-animal variability particularly in the M279 treated group. Measurements of tumour volume from immunofluorescent stained sections (**Figure 5A**), showed a significant decrease in M279 treated animals compared to both PBS and TKP treated groups (**Figure 5B**).

### 3.6 Inhibition of Anti-Inflammatory Macrophages/Microglia in An Intracardiac Brain Metastasis Model

**Figure 6:**
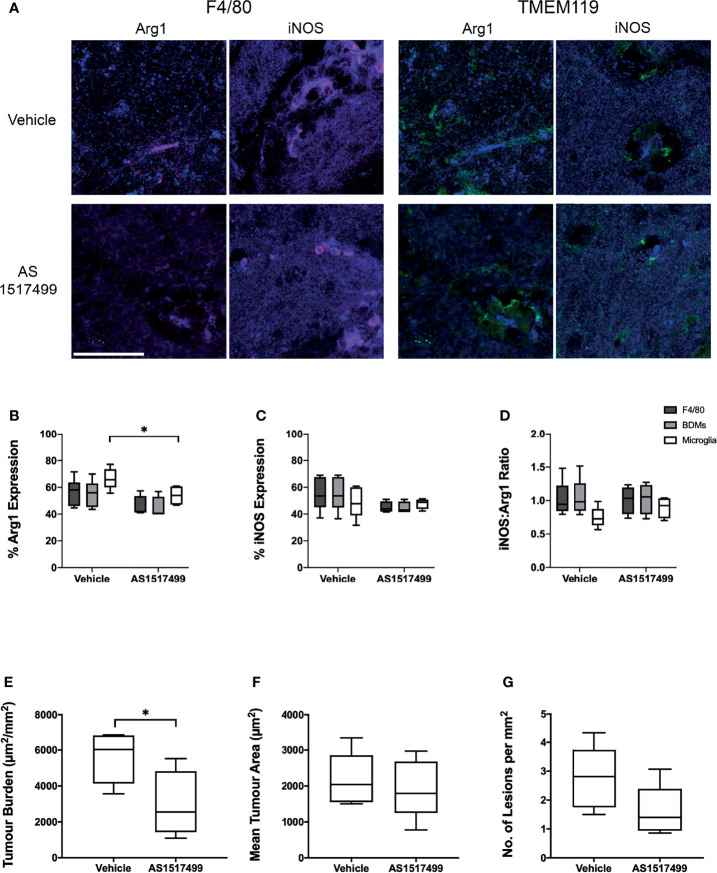
Effect of STAT6 Inhibition in BALB/c mice injected intracardially with 4T1-GFP cells. **(A)** Representative immunofluorescence images from vehicle and AS1517499 treated mice depicting co-expression of F4/80 (red, left panel) with Arg1 and iNOS (blue), and TMEM119 (green, right panel) with Arg1 and iNOS (blue). Scale bar = 40 µm. **(B–D)** Graphs showing **(B)** expression of Arg1, **(C)** expression of iNOS and **(D)** the ratio of iNOS : Arg1 expression in all F4/80+ cells (dark grey bars), microglia (TMEM119+F4/80+ cells, light grey bars) and BDM (TMEM119-F4/80+ cells, white bars) for vehicle (n = 6) and AS1517499 (n = 5) treated mice. **(E–G)** Graphs showing **(E)** tumour burden measured as total area of metastases divided by total tissue area analysed, **(F)** average tumour area, and **(G)** average number of metastases per square mm of tissue analysed. Data shown as box and whisker plots depicting full range of data points. *p < 0.05.

Finally, the effect of inhibiting the anti-inflammatory phenotype of BDM/microglia was determined in animals injected intracardially with 4T1-GFP cells and treated with either the STAT6 inhibitor AS1517499 or vehicle. *In vitro* culture of 4T1-GFP cells with AS1517499 showed no negative effects on tumour cell growth or viability (**Figure S5**). Immunostaining for F4/80 and TMEM119 with Arg1 or iNOS (**Figure 6A**) demonstrated a decrease in Arg1 expression in TMEM119+F4/80+ microglia in the AS1517499 treated group compared to the vehicle control group (p < 0.05; **Figure 6B**). Arg1 expression in total F4/80 and BDM also demonstrated a similar trend (p = 0.0667 for both). No significant changes were observed in iNOS expression (**Figure 6C**) or the ratio of iNOS : Arg1 expression (**Figure 6D**) in each cell population. Tumour burden was found to be significantly lower in the AS1517499 treated group (3000 ± 800 µm^2^/mm^2^) compared to the vehicle controls (5600 ± 550 µm^2^/mm^2^; p < 0.05; **Figure 6E**). Whilst the average lesion area was not significantly different between groups (**Figure 6F**), a trend towards a decrease in the number of lesions per mm^2^ was evident (p = 0.075, **Figure 6G**).

## 4 Discussion

Macrophages are known to impact systemic tumour progression and extracranial metastasis, and have also been implicated in glioma and breast cancer brain metastasis (4, 7, 13, 37, 38). There is mounting evidence that macrophages/microglia, and their different phenotypes, play an important role in brain metastasis progression, with an increasing number of studies suggesting a role for the anti-inflammatory phenotype specifically. In this study, initial *in vitro* experiments demonstrated that macrophage phenotype affects migration of metastatic breast cancer cells, with anti-inflammatory phenotype (IL-4-stimulated) leading to increased migration compared to pro-inflammatory phenotype (LPS-stimulated). Subsequent *in vivo* studies demonstrated that infiltration of blood-derived myeloid cells (predominantly macrophages; BDM) into brain metastases increased over time, and with an increasingly anti-inflammatory phenotype. Moreover, suppression of the anti-inflammatory phenotype in BDM, either through BDM specific KLF4 knock-out or antibody blockade of CSFR1, resulted in a significant reduction in brain metastasis growth. Finally, inhibition of the anti-inflammatory microglial phenotype *via* STAT6 inhibition, in a model of haematogenously disseminated brain metastases, also significantly reduced brain tumour burden. Together these data suggest that modulation of both BDM and microglial phenotype towards a pro-inflammatory profile has substantial therapeutic potential in brain metastasis.

Our *in vitro* work has demonstrated that an anti-inflammatory macrophage phenotype promotes breast cancer cell invasiveness. Previous work by Green et al. demonstrated an increase in colorectal (CT26) tumour cell migration and invasiveness in the presence of unstimulated RAW 264.7 macrophages or their conditioned media, as well as increases in transcription of genes associated with increased tumour aggressiveness (39). However, no information on the phenotype of the macrophages was reported. Macrophage TNF and TGFβ1 present within co-cultures have also been shown to increase the migration of MDA-231 breast cancer cells in a 3D culture model through MMP1 and MT1-MMP, respectively (40). This finding conflicts with our results as TNF is known to be a pro-inflammatory cytokine. However, the phenotype of activated macrophages is complex, potentially expressing a combination of pro- and anti-inflammatory markers and cytokines, dependent upon the specific environment surrounding the macrophages (10).

Our initial *in vivo* studies indicated significant recruitment of BDM to brain metastases, which not only increased over time but also became more anti-inflammatory in phenotype, with a decrease in the expression of iNOS relative to Arg1. It is important to recognise, however, that the phenotype of macrophages/microglia do not fall into discrete classes of pro-inflammatory or anti-inflammatory, but exist on a spectrum where both pro- and anti-inflammatory phenotype markers may be expressed within the same cell, or population of cells, but to differing degrees (10, 41). Our previous work demonstrated that anti-inflammatory macrophages and microglia, expressing Arg1 and the mannose receptor MRC-1 (CD206), are present within brain metastases and that these markers also tend to increase as the metastases progress (13). The current study builds on that work by differentially looking at the phenotype of BDM specifically, rather than the mixed population of BDM/microglia. Rippaus et al. previously evaluated the phenotype of macrophages and microglia in parenchymal and dural brain metastases. Their findings showed that BDM and microglia in parenchymal metastases have a more anti-inflammatory phenotype with a decrease in iNOS expression and an increase in CD206 expression, and that there is a phenotypic difference in BDM between dural and parenchymal metastases (12). In that study, microglia and BDM were identified based on relative expression of macrophage specific markers, which may not stratify BDM and microglia entirely. To address this potential limitation, and further assess the phenotype of BDM recruited to metastases, we have used a model that specifically expresses GFP in myeloid cells. Whilst the GFP expression occurs in all myeloid cells, we have found that in the brain metastasis model used here the contribution of non-BDM cells (GFP+F4/80-) to the overall GFP+ infiltrating population was <10%. Thus, our results predominantly reflect infiltrating BDM, although we cannot exclude a minor contribution from granulocytes. This finding is consistent with previous studies, in which the major infiltrating population in parenchymal brain metastases were CD11b+F4/80+ cells (12, 42). Overall, the results of this study are consistent with previous reports and demonstrate increasing infiltration of anti-inflammatory BDM, specifically, over time.

Given the increasingly anti-inflammatory phenotype of recruited BDM in the above study, our next aim was to determine the impact of this phenotype on brain metastasis growth. The transcription factor KLF4 has been shown to be a key regulator of macrophage polarization in models of both prostate (43) and breast cancer (6), and is activated through STAT6 signaling; this, in turn, is triggered through IL-4 signaling and participates in limiting the pro-inflammatory response (44). Here, we have shown that in KLF4 knock-out animals the anti-inflammatory response of BDM, but not brain-resident microglia, is significantly suppressed, and that this is associated with a reduction in brain metastasis growth. Subsequent studies, using the more clinically relevant antibody-mediated inhibition of anti-inflammatory BDM, further confirmed the above finding, and together these data support the notion that anti-inflammatory BDM recruited to brain metastases are pro-tumorigenic. These results are in accord with our previous work, in which mannosylated clodronate liposomes were used to deplete the total anti-inflammatory BDM/microglia population in an intracerebral brain metastasis model, using 4T1-GFP cells in BALB/c mice. In that work, we observed a significant decrease in anti-inflammatory BDM/microglia, together with a reduction in tumour growth (13).

In accord with our findings, the antibody used in our studies, M279, which does not readily cross the BBB, has previously been shown not to alter the resident microglial phenotype when administered systemically (26, 27), and to inhibit anti-inflammatory monocyte/macrophage activation without affecting pro-inflammatory activation (26). The above findings are also in agreement with previous studies showing that inhibition targeting the CSF-1R pathway shifted the phenotype of activated tumour-associated macrophages (TAMs) towards a more pro-inflammatory phenotype, resulting in reduced tumour growth in glioblastoma models (7, 8), as well as in extracranial models of breast and cervical cancer (45). Moreover, it has also been shown that the addition of CSF-1R pathway inhibition to adoptive cell therapy in preclinical melanoma models can improve the anti-tumour response (46). However, those studies used small molecule inhibitors of CSF-1R, such as BLZ495 and PLX3397, which are known to be BBB penetrant. Interestingly, in contrast to the current work, studies with such molecules have shown an overall decrease in macrophage and/or microglial recruitment (7, 47), although a decrease in M2 (anti-inflammatory) genes has also been observed, which is consistent with our work (7). As M279, could not act directly on microglia in our study, the observed increase in microglial recruitment may reflect paracrine signaling from other cells within the microenvironment (48, 49).

Interestingly, whilst others have shown that TKP is capable of attenuating activation of both BDM and microglia (24, 25), in the current study we found no significant changes in either BDM/microglia numbers or phenotype following TKP treatment. These differences may reflect the models used in those previous studies, which were predominantly pro-inflammatory (e.g. experimental autoimmune encephalomyelitis (EAE), spinal cord injury and intracerebral ischemic hemorrhage) (24, 25, 50) and, consequently, represent an entirely different immune environment to cancer. At the same time, the relatively short time-course of the treatment and inter-animal variability may have reduced sensitivity to the effects of TKP in the current study; there is some minor suggestion of a movement towards reduced numbers of both BDM/microglia and pro-inflammatory phenotype in the TKP treated group when compared to the controls, and a possible increase in tumour volume. Nevertheless, none of these changes reach significance and further work would be required to confirm or refute these observations.

Finally, we wanted to test the hypothesis that inhibition of the anti-inflammatory macrophage/microglial phenotype reduces brain metastasis volume in a more physiologically representative model, induced *via* haematogenous tumour cell dissemination to the brain. In this study, treatment with the STAT6 inhibitor AS1517499 was associated with a significant decrease in Arg1 expression in microglia and a significant reduction in brain tumour burden. Notably, AS1517499 did not reduce 4T1-GFP cell proliferation or viability *in vitro* (**Figure S7**), indicating that the observed reduction in tumour growth does not reflect a direct effect of AS1517499 on the tumour cells’ viability themselves. Nevertheless, we cannot exclude an effect of AS1517499 on the tumour cells’ transcriptional profile, which could in turn impact tumour progression through changes in secreted factors and paracrine interactions with the microenvironment. Although there was also a trend towards reduced Arg1 expression in BDM, this did not quite reach significance. Together, these data suggest that the anti-inflammatory phenotype of brain-resident microglia may also be pro-tumorigenic and, hence, contribute to brain metastasis progression. Similarly, previous studies have demonstrated that STAT6 inhibition with AS1517499 suppresses the anti-inflammatory macrophage phenotype *in vitro*, whilst leading to reduced primary breast tumour volume and lower incidence of liver metastasis *in vivo* (6).

Together the above findings suggest that targeting the anti-inflammatory phenotype of microglia/macrophages may provide an effective treatment strategy for brain metastasis, most likely in combination with other currently available therapies. Drugs targeting the CSF-1 signalling pathway are currently in development and many are in clinical trial. Several of these drugs have shown great promise, but are susceptible to acquiring tumour resistance (51). Inhibition of the CSF-1 pathway can be overcome by IL-4 stimulation of TAMs within the tumour microenvironment. Quail et al. have demonstrated that this IL-4 stimulation leads to IGF-1 secretion by TAMs, which in turn activates IGF-1R and PI3K signalling in tumour cells driving tumour relapse (8). Those authors also demonstrated that regrowth could be curbed by adding inhibitors that target the IGF-1R and PI3K pathways as well as targeting STAT6 directly after the initial CSF-1R targeted therapy (8). Moreover, studies by Pradel et al. of Emactuzumab (Humanized IgG1 anti-CSF-1R antibody) have shown that *in vitro* IL-4 stimulation can overcome CSF-1R inhibition in macrophages (52). Thus, combining CSF-1 and IL-4 pathway inhibition with additional therapies may be important and have a synergistic effect. CSF-1/CSF-1R blockade has also been shown to reprogram tumour associated macrophages, and enhance the response to checkpoint immunotherapy in a pancreatic ductal adenocarcinoma model (53). A preclinical study of CSF-1R inhibition with BRAF inhibitors demonstrated that these therapies complement each other and produce a robust anti-tumour effect (46). Finally, Quail et al. have shown that AS1517499 inhibition of STAT6 in combination with CSF-1R inhibition reduces CSF-1R resistant glioblastoma regrowth (8).

The current work has focused predominantly on systemically derived BDMs. However, the results of the final study suggest that microglia may also influence metastasis progression. In support of this notion, whilst not significant, we did note trends towards changes in inflammatory marker expression in microglia that followed those observed in BDM in both the KLF4 knockout and pharmacological inhibition studies. These observations may reflect paracrine signalling between BDM and microglia, and possibly other cells within the environment, such as astrocytes and endothelial cells. The microenvironment of brain metastases is complex, with many different cell types potentially contributing to, or inhibiting, progression (48, 49). Additional studies where both populations are inhibited, as well as microglia specifically, will be invaluable in determining the exact contributions of each macrophage population, and how they interact with other components of the tumour microenvironment.

In conclusion, inhibition of the anti-inflammatory phenotype in blood-derived macrophages and/or brain-resident microglia, through targeting of CSF-1R or STAT6/KLF4, reduces metastasis growth in the brain. This work provides strong support for the concept that modulating the inflammatory phenotype of both blood-derived macrophages and microglia towards a more pro-inflammatory phenotype may have significant therapeutic benefits in brain metastasis, particularly in combination with other therapies, and may significantly improve prognosis for patients with brain metastasis.

## Data Availability Statement

The raw data supporting the conclusions of this article will be made available by the authors, without undue reservation.

## Ethics Statement

The animal study was reviewed and approved by the University of Oxford Clinical Medicine Ethics Review Committee and approved by the UK Home Office (Animals [Scientific Procedures] Act 1986).

## Author Contributions

VE and NS designed the research. VE, MP, VJ, HS, and KA performed the research. VE performed the analysis. KA assisted with model development. JL contributed to statistical analysis. VE and NS wrote the manuscript. All authors contributed to reviewing the work and revising it critically for important intellectual content. All authors approved the version to be published and agree to be accountable for all aspects of the work.

## Funding

This work was supported by an MSCA fellowship (MSCA-IF-EF- ST-654985) from the European Commission to VE, Cancer Research UK (C5255/A15935), Breast Cancer Now (2016NovPRB31), the Engineering and Physical Sciences Research Council (EP/L 024012/1), and a Cancer Research UK studentship to KA.

## Conflict of Interest

Author MP is currently employed by OxSonics Ltd.

The remaining authors declare that the research was conducted in the absence of any commercial or financial relationships that could be construed as a potential conflict of interest.

## Publisher’s Note

All claims expressed in this article are solely those of the authors and do not necessarily represent those of their affiliated organizations, or those of the publisher, the editors and the reviewers. Any product that may be evaluated in this article, or claim that may be made by its manufacturer, is not guaranteed or endorsed by the publisher.
