# Supplementary material for: Inhibition of Anti-Inflammatory Macrophage Phenotype Reduces Tumour Growth in Mouse Models of Brain Metastasis
Source: Front Oncol. 2022 Mar 10;12:850656. doi: 10.3389/fonc.2022.850656 (PMC8960618; doi:10.3389/fonc.2022.850656)
Supplement: Supplementary file 1 [file DataSheet_1.pdf]

# *Supplementary Material*

## 1 Supplementary Figures

### 1.1 Figure S1

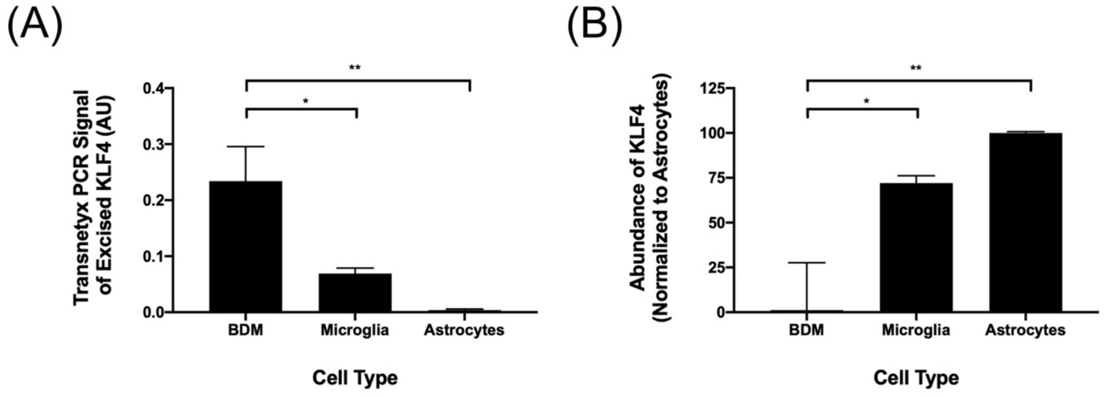


Genotyping of $LysM^{Cre}$ $KLF4^{(fl/fl)}$ mice. Astrocytes and microglia were isolated from brain cultures derived from 3-day old pups. BDM were isolated from bone marrow derived from adult mice. The cells were pelleted into genotyping plates provided by Transnetyx and shipped to their facility for analysis. PCR analysis was conducted to identify the excised version of the floxed KLF4 gene in all samples. (A) Graph showing acquired PCR signal of excised KLF4 gene in all cell types. (B) Graph showing abundance of the intact version of the floxed KLF4 gene in all cells types (normalized to astrocytes). N = 4 for each cell type. Data are shown as mean ± SEM. One-way ANOVA test, with Tukey's post-test. * $p<0.05$, ** $p< 0.01$.

## 1.2 Figure S2

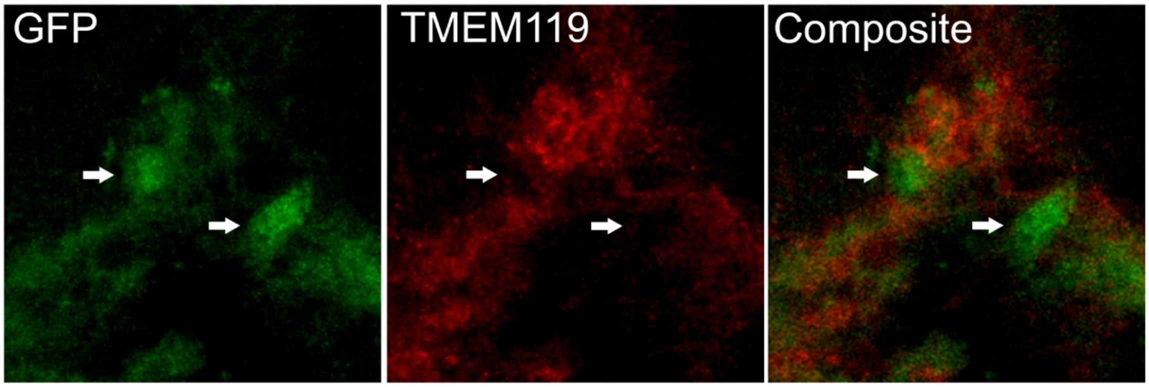


Immunofluorescent staining of TMEM119 in Lys-GFP-ki mice. Arrows indicate GFP+ cells (BDM) that do not show co-localization with TMEM119, supporting the use of TMEM119 as a microglial-specific marker in this model.

## 1.3 Figure S3

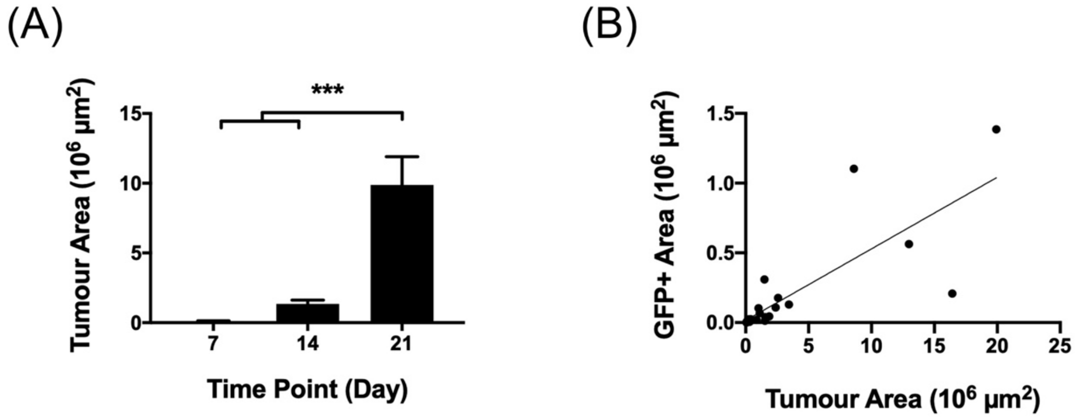


Tumour growth and correlation with BDM/macrophage infiltration. (A) Graph showing progression of tumour area with time. (B) Graph showing correlation between tumour area and GFP positive area, indicating a linear relationship between infiltration of BDM/macrophages and tumour growth ($r^2 = 0.6398$, $p < 0.0001$). N = 9 at day 7, n = 9 at day 14 and n = 10 at day 21. Data shown as mean ± SEM. *** $p < 0.001$

## 1.4 Figure S4

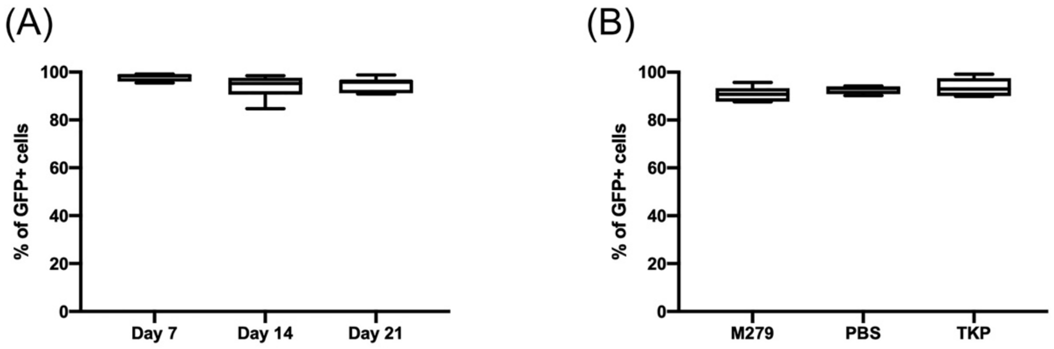


Quantification of BDM contribution to myeloid cell infiltration within EO771 tumour in Lys-GFP-ki mice. (A) Percentage of GFP+ cells that are also F4/80+ (BDM) within tumours at days 7, 14 and 21 after tumour induction ($p > 0.2$, One-way ANOVA). (B) Percentage of GFP+ cells that are also F4/80+ (BDM) within tumours treated with TKP, PBS or M279 ($p > 0.3$, One-way ANOVA). Data shown as box and whisker plots depicting full range of data points.

## 1.5 Figure S5

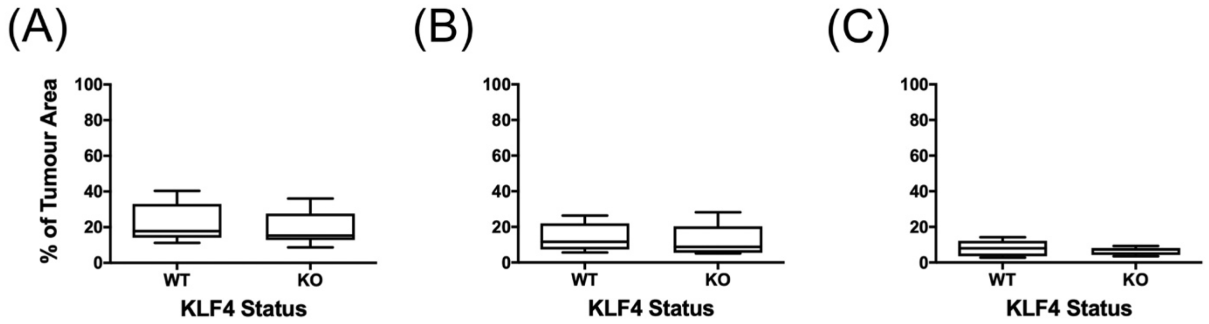


Phagocyte infiltration in metastases for KLF4 wild type (n = 9) and KO (n = 8) mice. (A) Graph showing F4/80+ staining as a percentage of tumour area. (B) Graph showing microglial (TMEM119+ F4/80+) staining as a percentage of tumour area. (C) Graph showing BDM (TMEM19- F4/80+) staining as a percentage of tumour area. Data are shown as box and whisker plots depicting full range of data points. No significant differences were found between groups.

## 1.6 Figure S6

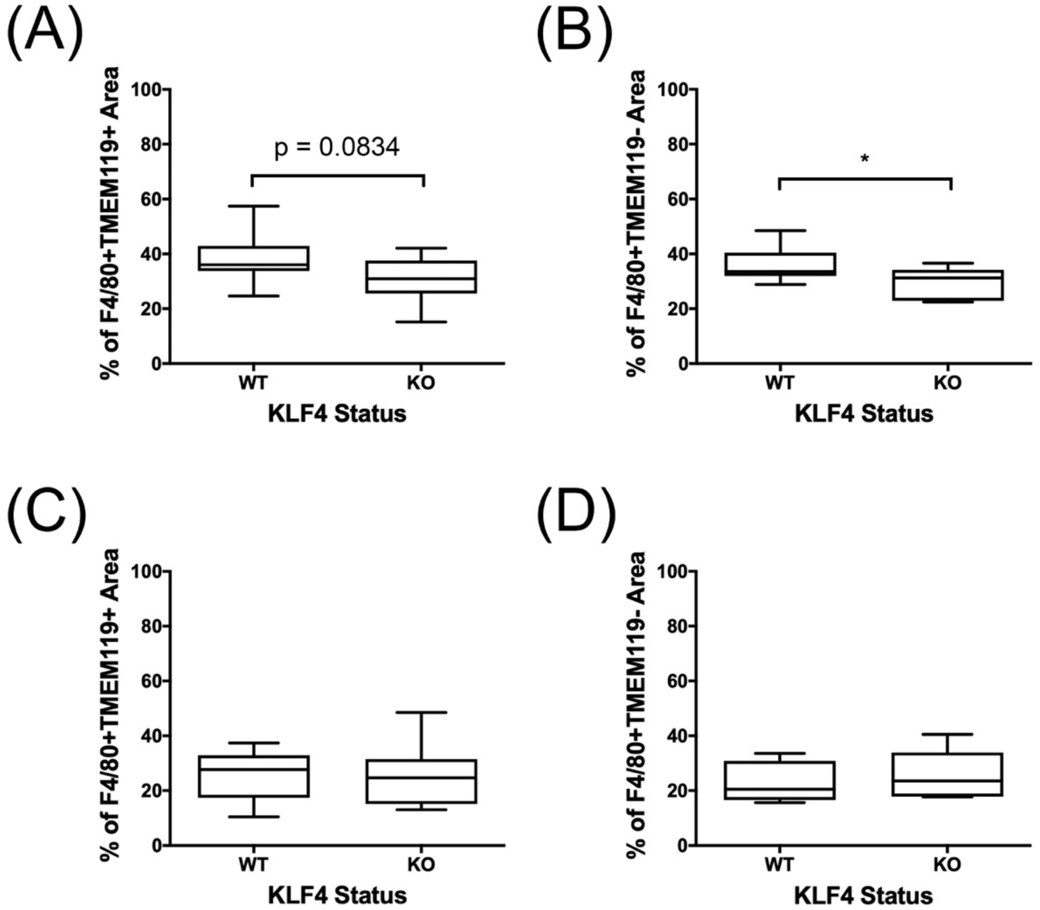


Phagocyte phenotype in metastases from KFL4 wild type (n = 9) and KO (n =8) mice. (A-B) Graphs showing Arg1+ staining as a percentage of (A) microglial (F4/80+TMEM119+) area and (B) BDM (F4/80+TMEM119-) area. Graphs showing iNOS+ staining as a percentage of (C) microglia (F4/80+TMEM119+) area and (D) BDM (F4/80+TMEM119-) area. Data are shown as box and whisker plots depicting full range of data points. * $p < 0.05$.

## 1.7 Figure S7

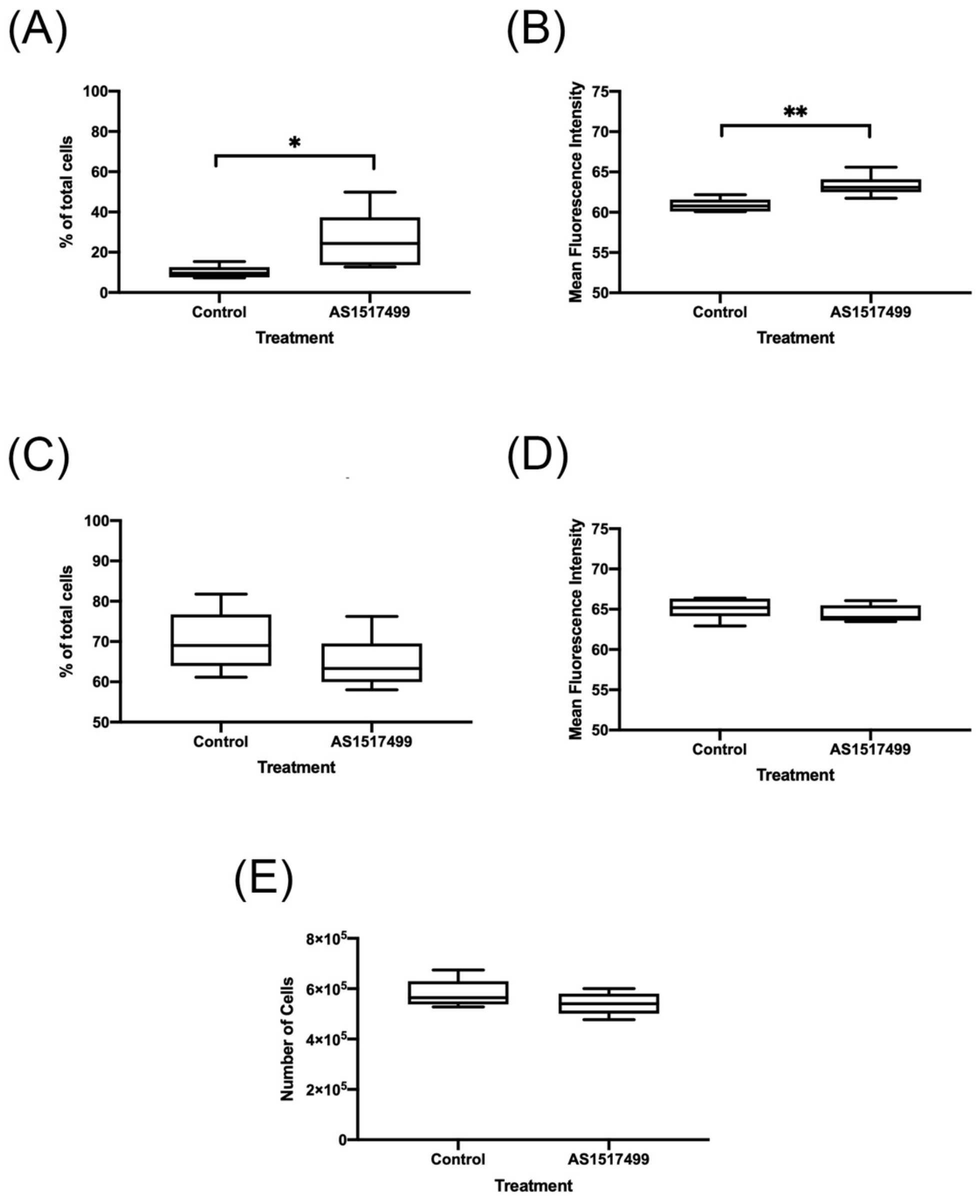


*In vitro* culture of 4T1-GFP cells with AS1517499. Cells were seeded into 6-well plates and allowed to adhere before being incubated with AS1517499 or vehicle for a period of 48 hours. Cells were stained for cleaved Caspase 3 and Ki67 to assess apoptosis and proliferation. Graphs depicting (A) percentage of cells that are Ki-67+, (B) mean fluorescent intensity of Ki-67, (C) percentage of cleaved Casp3+ cells, (D) mean fluorescent intensity of cleaved Casp3, and (E) total number of 4T1-GFP cells. Data were analysed using an unpaired student t-test and are shown as box and whisker plots depicting full range of data points. * $p < 0.05$, ** $p < 0.01$.

# 2 Supplementary Tables

## 2.1 Table S1 – Table of antibodies used for immunofluorescent staining

| Marker | Primary Antibody (Dilution) | Secondary Antibody (Dilution) or Tagged Fluorophore | Fluorophore(s) for Biotinylated Secondary Antibodies |
|---|---|---|---|
| F4/80 | Anti-mouse F4/80, Raised in Rat, Abcam (1:50) | Anti-Rat IgG, Biotin Tagged, Vector Labs (1:100) | Cy3 – Streptavidin (1:30) |
| | | Anti-Rat IgG, Texas Red Tagged, Vector Labs (1:100) | |
| | | Anti-Rat IgG, Dylight 488 Tagged, Vector Labs (1:100) | |
| iNOS | Anti-mouse iNOS, Raised in Rabbit, Abcam (1:100) | Anti-Rabbit IgG, Texas Red Tagged, Vector Labs (1:100) | |
| | Anit-mouse iNOS, Raised in Mouse, Abcam (1:100)* | Anti-Mouse IgG, Biotin Tagged, Vector Labs (1:100) | Dylight 405-Streptavidin (1:100) |
| Arg1 | Anti-mouse Arg1, Raised in Mouse, Santa Cruz (1:50)* | Anti-Mouse IgG, Biotin Tagged, Vector Labs (1:100) | AMCA-Streptavidin (1:30);<br>Dylight 405-Streptavidin (1:100) |
| | | Anti-Mouse IgG, Texas Red Tagged, Vector Labs (1:100) | |
| TMEM119 | Anti-mouse TMEM119, Raised in Rabbit, Abcam (1:100) | Anti-Rabbit IgG, Texas Red Tagged, Vector Labs (1:100) | |
| | | Anti-Rabbit IgG, Alexa Fluor 647 tagged, Life Technologies (1:100) | |

*Mouse IgG blocking was performed using the Mouse-on-Mouse staining kit (Vector Labs) according to the manufacturer's recommendations

## 2.2 Table S2 – Sample Size Calculation

| | | | | | |
|---|---|---|---|---|---|
| **SD of Pilot Data ($\mu m^3$)** | 2.78E+09 | | | | |
| **Effect size ($\mu m^3$)[1]** | 5.13E+09 | | | | |
| | | | | | |
| **Confidence Level** | **0.05** | **0.01** | **0.001** | | |
| **$Z_a$** | 1.96 | 2.5758 | 3.2905 | | |
| | | | | | |
| **Power** | **80** | **85** | **90** | **95** | |
| **$Z_{(1-b)}$** | 0.8416 | 1.0364 | 1.2816 | 1.6449 | |
| | | | | | |
| **Sample size required** | | | | | |
| | | **Power** | | | |
| | **Confidence level** | **80** | **85** | **90** | **95** |
| | **a = 0.05** | 5 | 6 | 7 | 8 |
| | **a = 0.01** | 7 | 8 | 9 | 11 |
| | **a = 0.001** | 11 | 12 | 13 | 15 |
| | | | | | |

[1] 20% of average tumour volume

Calculation based on methods described by Kadam and Bhalerao (1)

## 3 SI References


1. P. Kadam, S. Bhalerao, Sample size calculation. Int. J. Ayurveda Res. 1, 55–57 (2010).
